# Prevalence and determinants of antenatal depression and its association with intimate partner violence: a cross-sectional study at Hospital Melaka, Malaysia

**DOI:** 10.3389/fpsyt.2024.1466074

**Published:** 2024-11-22

**Authors:** Sethuckarasi Narayanan, Fairuz Nazri Abd Rahman, Rosnah Sutan, Gayathri K. Kumarasuriar, Andrew Kah Seong Leong

**Affiliations:** ^1^ Department of Psychiatry, Faculty of Medicine, Universiti Kebangsaan Malaysia, Kuala Lumpur, Malaysia; ^2^ Department of Psychiatry and Mental Health, Hospital Melaka, Melaka, Malaysia; ^3^ Child and Adolescent Psychiatric Unit, UKM Specialist Children’s Hospital, Kuala Lumpur, Malaysia; ^4^ Department of Community Health, Medical Faculty, Universiti Kebangsaan Malaysia, Kuala Lumpur, Malaysia; ^5^ Department of Obstetrics and Gynecology, Hospital Melaka, Melaka, Malaysia

**Keywords:** depression, postpartum, domestic violence, emotional abuse, pregnancy, intimate partner violence

## Abstract

**Objective:**

This study aimed to assess the determinants of antenatal depression and its relation to intimate partner violence (IPV) among pregnant women at Hospital Melaka, Malaysia.

**Methods:**

A cross-sectional study was conducted between February 1 and March 31, 2024, with 370 pregnant women recruited through convenience sampling. Inclusion criteria were Malaysian citizenship, age above 18 years, and ability to read and comprehend Malay. Data collection involved self-reported sociodemographic questionnaires, the Edinburgh Postnatal Depression Scale (EPDS), and the WHO Multicountry Study on Women’s Health and Life Events Questionnaire.

**Results:**

The prevalence of antenatal depression was 8.4%. IPV was reported by 64.1% of participants, with 54.6% experiencing controlling behavior, 30.0% emotional violence, 2.4% physical violence, and 3.5% sexual violence. Bivariate analysis showed that emotional violence (p < 0.001), physical violence (p < 0.001), sexual violence (p < 0.001), and hospitalization (p = 0.006) were significantly associated with an increased risk of antenatal depression. Multivariable logistic regression revealed that women receiving outpatient care had significantly lower odds of developing antenatal depression compared to hospitalized women (adjusted OR 0.262, 95% CI 0.100–0.683; p = 0.006). Women who experienced sexual violence were 18 times more likely to develop antenatal depression (adjusted OR 18.761, 95% CI 3.603–97.684; p < 0.001).

**Conclusion:**

The study highlights the need for healthcare workers to recognize risk factors for antenatal depression, particularly IPV.

## 1 Introduction

Pregnancy is a significant life event accompanied by psychological and physiological changes that make pregnant women more vulnerable to the onset or recurrence of mental disorders. Antenatal depression is one of the most prevalent psychiatric disorders, severe negative impacts on both the mother’s and the infant’s health (1). Antenatal depression prevalence was reported to range from 15 to 65% globally (2). According to a local study, the prevalence of antenatal depression in Pahang and Selangor, Malaysia was 12% (3). Depression is a mental health condition that manifests as significant mood changes and is linked to distress with or without functional impairment. Depression can range in severity from mild to severe with affected people tending to have a reduced quality of life and an increased risk for suicide (4).

It has been found that antenatal depression has long-lasting adverse effects on pregnant mothers, their offspring, and their families. According to several studies, depression during pregnancy is linked to high default rates in prenatal clinic attendance and fetal growth abnormalities. Preeclampsia and complicated labours are also more prevalent in women with antenatal depression (4). Studies revealed that, on average, 39% of women who experienced antenatal depression also experienced postnatal depression (5). Another study found that antenatal depression increases the risk of postnatal depression by 20 times (6). Prevention of antenatal depression is essential because, without proper treatment and management, depression symptoms in pregnancy may persist after childbirth.

A Malaysian paper has mentioned that previous studies have identified some risk factors for depression during pregnancy, such as intimate partner violence (IPV), lack of partner or social support, personal history of mental illness, and unplanned pregnancy (7). There is a significant and positive association between IPV and antenatal depression among pregnant women. Antenatal depression was 2.5 times as prevalent in pregnant mothers who had experienced IPV (8).

The term “intimate partner violence” is now preferred over “domestic violence” (9). IPV includes physical, sexual, and emotional violence as well as controlling behaviour committed by a male intimate partner, whether cohabiting or not, in the present or in the past. The WHO Multi-country Study on Women’s Health and Domestic Violence provides operational definitions of violence. It defines physical violence by an intimate partner as acts including slapping or having objects thrown at the victim, pushing or shoving, hitting with a fist or object, kicking, dragging, beating, choking, burning, and threats or actual use of weapons such as guns or knives. Sexual violence by an intimate partner is defined as the victim being physically forced into unwanted sexual intercourse, engaging in sexual intercourse out of fear of the partner, and being forced to perform degrading or humiliating sexual acts. Emotional abuse by an intimate partner includes being insulted or made to feel bad, belittled or humiliated in front of others, deliberately scared or intimidated, and threatened with harm to someone the victim cares about. Controlling behaviors include efforts to restrict the victim’s access to friends and family, insisting on knowing her whereabouts at all times, treating her indifferently, displaying anger when she interacts with other men, being suspicious of her fidelity, and expecting her to ask for permission before seeking healthcare.

According to a WHO Study (2005), the lifetime prevalence of physical or sexual violence among women in ever-partnered relationships ranged from 30 to 60%. More than 5% of women had been physically mistreated during at least one pregnancy and over 90% of these women suffered maltreatment at the hands of the child’s biological father. The study found that violence increased during pregnancy. The Malaysian Women’s Aid Organization carried out the largest national research in 2000 with 1221 respondents from across the country. According to this survey, 36% of intimate relationship abuse was physical, affecting both married and unmarried couples.

Using the WHO Women’s Health and Life Experiences Questionnaire, Rashidah Shuib et al. (10) conducted a landmark study in 2013 on domestic abuse and women’s well-being in Malaysia. Although there was an 8% frequency of violence against women, the study suggested underreporting may still exist. While violence affects women of all ages, studies have found that pregnant women and low-income individuals are at greater risk for IPV (11). A study done in Southern Ethiopia reported a prevalence of 30.2% IPV reported among women with antenatal depression (12).

In the study by Nasreen et al. (3), determinants were identified by interviewing women about their socioeconomic backgrounds. This study uses the WHO Women’s Health and Life Experiences Questionnaire. It was developed to measure women’s health and IPV experiences. This study also supported that maternal depressive symptoms were positively associated with IPV, which was at 3% (3). Additionally, studies have indicated that women who experienced IPV during the antenatal and postnatal period are more likely to experience depression and post-traumatic stress disorder after the delivery and that these issues may influence how mothers respond to their children. One study in Zimbabwe reported that women who experienced intimate partner violence were 2.5 times more likely to have antenatal depression than those who did not (13).

Based on the existing literature, we hypothesize that pregnant women who experience IPV are more likely to develop antenatal depression. Furthermore, we hypothesize that different types of IPV (controlling behavior, emotional, physical, and sexual violence) are significantly associated with varying levels of antenatal depression.

Studies on the association between IPV and antenatal depression in Malaysia are limited. Despite the growing recognition of antenatal depression and its association with IPV, there are still gaps in local research. While Nasreen et al. (31) and Arifin et al. (5) have provided valuable insights into the prevalence and risk factors of antenatal depression in Malaysia, there remains a significant gap in understanding the long-term impact of these conditions on both mother and child. Many studies, including this current research, use cross-sectional designs to efficiently identify associations between antenatal depression and IPV within a limited timeframe. However, these studies serve as a crucial first step in laying the groundwork for future research that can explore the causal relationships and longitudinal effects of antenatal depression and IPV, which are not easily captured in a single time-point analysis. Furthermore, while there has been substantial research on the physical health consequences of pregnancy complications like hyperemesis gravidarum, as examined by Azlan et al. (7), there is limited exploration of the psychological dimensions of these conditions.

Thus, the knowledge gap is not in the prevalence data alone but in the deeper understanding of how these mental health issues evolve and affect outcomes such as maternal mental health. This study aims to contribute to the existing body of local research but also highlights the urgent need for future longitudinal studies in Malaysia to follow these women beyond pregnancy and into the postpartum period to assess long-term mental health outcomes. This study aims to evaluate the determinants of antenatal depression and its relationship with IPV among pregnant women attending the Obstetrics and Gynaecology (O&G) clinic at Hospital Melaka. Specifically, the objectives are to assess the prevalence of antenatal depression, measure the prevalence of different forms of IPV, and investigate the association between IPV and antenatal depression in this population.

## 2 Methodology

### 2.1 Study design, setting, and sample

This cross-sectional study was conducted in the Hospital Melaka O&G Department. Melaka is located in the southern region of Peninsular Malaysia, bordered by Negeri Sembilan to the north and Johor to the south. As of the most recent estimates, Melaka has a population of approximately 1 million people (14). This population is diverse, comprising different ethnics, and occupations.

Hospital Melaka serves a wide area, including the Melaka Tengah, Jasin, and Alor Gajah districts in Melaka. Additionally, it extends its services to the southern part of Negeri Sembilan and the northern region of Johor. This extensive coverage highlights the hospital’s role as a key medical facility in the region, providing comprehensive healthcare services to a large and diverse population (15). In 2023, more than 18,000 patients attended the antenatal clinic in Hospital Melaka and over 14,000 patients were admitted to the antenatal wards yearly.

Hospital Melaka plays a pivotal role as the primary site for gathering data on antenatal depression and its association with IPV. By serving as the research site, Hospital Melaka is an ideal location to explore the determinants of antenatal depression. Understanding the determinants of antenatal depression in the local context, particularly its association with IPV, can help in developing targeted interventions to support at-risk women.

The participants were recruited from both the outpatient clinic and the inpatient wards. The participants were pregnant women who come for antenatal checkups in the clinic, as well as patients admitted to the antenatal wards.

The sample size was calculated using the prevalence of 32.0% IPV reported among antenatal depression, as this study aims to assess the association between antenatal depression and IPV among pregnant women and determine its factors (12). Therefore, the sample size was calculated using the occurrence of IPV among women with antenatal depression. Sample size calculations for other factors were also performed, and the highest calculated sample size was chosen.

Assuming a 95% confidence interval (CI), the minimum sample size required for the present study was 335 cases of pregnant women. With an additional non-response of 10%, the total sample size needed was 369.

The inclusion criteria required participants to be Malaysian pregnant women aged 18 or older who can independently provide consent and who could read and comprehend the Malay language. Women were excluded if they were unable to participate in the interview due to active labor, reduced consciousness, or cognitive impairment, or if they were in the labor room or operating theatres.

### 2.2 Data collection

Participants were recruited using convenience sampling, and data was collected between February 1 2024 and March 31, 2024. They were recruited from both the outpatient antenatal clinic and the inpatient wards of Hospital Melaka. Outpatient participants were approached during their routine antenatal check-ups. Inpatient participants were approached during their stay in the antenatal ward, where they were either being monitored for pregnancy-related complications or preparing for delivery. The participants were at varying stages of gestation, but all were in the antenatal period. Data collection occurred in private settings to ensure confidentiality and comfort for the participants, regardless of their clinical situation.

Data collection was conducted by the first author to ensure consistency in approach. The participants were invited to join the study during their antenatal appointment dates. The first author provided the women with an Information Sheet and Consent Form. Those who consented to participate were brough into a private room within the clinic, away from their partners, and data was collected through a self-reported sociodemographic questionnaire. They were then given the self-administered EPDS to assess antenatal depression. Once the screening was complete, the first author conducted a face-to-face interview in the same private room using the WHO Multicountry Study on Women’s Health and Life Events Questionnaire. Similarly, data was collected at their bedside after the visiting hours to ensure privacy from the women’s partners, with the curtains drawn around the bed.

Participants required approximately 30 minutes to complete the questionnaires. Data collection was uneventful, with no incidents of distress caused by recalling past experiences while answering the questionnaires.

### 2.3 Ethical approval

This research was approved by the UKM Research and Ethics Committee (UKM PPI/111/8/JEP-2023-593) and the Malaysian Medical Research and Ethics Committee (MREC). The study has been registered under the National Medical Research Register Malaysia (NMRR-ID-23-03491-2RC) and received approval from the director of Hospital Melaka.

To ensure the confidentiality, all data extraction sheets will be coded and securely stored in a locked drawer. Hard copies of the study data will be retained for a period of 10 years, after which they will be securely destroyed.

### 2.4 Study instruments

#### 2.4.1 Sociodemographic data sheet

The participants’ social information was obtained using a questionnaire, which included questions on the age group, race, marital status, highest education level, occupation, household income, and history of mental illness. Clinical features were also collected, such as being inpatient or outpatient status, number of pregnancies, and diagnosis of the current pregnancy’s complications.

#### 2.4.2 Malay version of the Edinburgh postnatal depression scale

The EPDS is a validated, 10-item, self-rating scale used to measure antenatal and postnatal depression on a Likert scale from 0 to 3. The respondent selects the response that best reflects how she has been feeling over the past week.

The instrument has been validated for screening postpartum depression in Malaysia for showing a sensitivity of 72.7% and specificity of 95% at the cut-off 11.5 (16). It is a widely used screening tool for depression during the perinatal period. The cut-off for the Malay version of EPDS is ≥12 according to the Malaysian Clinical Practice Guidelines (CPG) on Management of Major Depressive Disorder (17). We used a cut-off point of ≥12 in this study.

#### 2.4.3 Malay version of the World Health Organisation women’s health and life experiences questionnaire

The WHO has developed the Women’s Health and Life Experiences to measure women’s health and IPV experiences (9). It was originally created for the WHO Multi-country Study on Women’s Health and Domestic Violence against Women. The questionnaire comprises 12 sections. In our study, we utilised Section 7 (Experiences of Partner Violence) to collect information on IPV, employing a variety of behaviour-specific questions to measure the prevalence of various types of violence.

The questionnaire was translated into Malay and validated to measure IPV among women in Malaysia by Saddki et al. (18). Construct validity and reliability assessment of the Malay version of the questionnaire was done on 20 specific items that measure four types of IPV act: (1) controlling behaviors (CB), (2) emotional violence (EV), (3) physical violence (PV) and (4) sexual violence (SV) which were the domains of interest. The Cronbach’s α values ranged from 0.767 to 0.858 across domains (18).

### 2.5 Statistical analysis

The data was analyzed using Statistical Package for Social Science (SPSS), licensed to Universiti Kebangsaan Malaysia. Data normality was assessed using the Kolmogorov-Smirnov test, which indicated that the dataset did not follow a normal distribution (p < 0.001). Therefore, non-parametric statistical methods were applied.

All questionnaires were checked for completeness, and no missing or out-of-range data were identified. Descriptive analysis was used to assess data distribution, while bivariate analysis (Chi-square) was conducted to examine associations between independent variables (age, race, marital status, occupation, income, education level, planned pregnancy, patient type, psychiatric illness, and intimate partner violence) and the dependent variable (antenatal depression).

A multivariable logistic regression model was employed to assess factors associated with antenatal depression, with IPV as the primary predictor. Other confounders included age, race, marital status, education level, occupation, pregnancy planning, and household income. Adjusted odds ratios (AORs) and 95% confidence intervals (CIs) were calculated, and significance was set at p < 0.05.

## 3 Results

A total of 384 pregnant women were approached; however, only 370 were successfully interviewed, and all the questionnaires were completed, resulting in a response rate of 96.4%. A total of 10 respondents did not complete the questionnaires as they were called for either an ultrasound scan or to be reviewed by the clinic doctor. Two respondents were excluded as they were not Malaysian citizens, one respondent was underage, and one refused to participate.

The sociodemographic characteristics of the respondents are shown in **Table 1**. The majority of the respondents were between 25 and 36 years of age. Among all the respondents, 98.9% were married, and 95.7% had attained at least a secondary education level. Employment status was nearly evenly split, with 54.1% employed and 45.9% unemployed. Among the respondents, 44.1% had planned pregnancy. Additionally, 44.1% of the patients were inpatients. When examining for psychiatric illnesses, 93.5% of the patients had no psychiatric illness. Among the respondents, 3.8% had depressive disorder, and 1.9% had anxiety disorder. Additionally, 72.1% had a total household income of less than RM4500.

**Table 1:** Sociodemographic characteristics of the respondents at Hospital Melaka.

Sociodemographic profile		N (370)	
		Count	%
Age	18-24	46	12.4%
	25-29	114	30.8%
	30-34	110	29.7%
	35 and above	100	27.0%
Race	Malay	319	86.2%
	Chinese	21	5.7%
	Indian	15	4.1%
	Others	15	4.1%
Marital Status	Single	1	0.3%
	Married	366	98.9%
	Divorced	3	0.8%
	Spouse deceased	0	0.0%
Occupation	Employed	200	54.1%
	Unemployed	170	45.9%
Income	< RM 2500.00	133	35.9%
	RM 2501.00 – RM4500.00	134	36.2%
	RM 4501.00 – RM 6500,00	63	17.0%
	RM 6501.00 – RM 8500.00	25	6.8%
	RM 8501.00 – RM 10500.00	8	2.2%
	> RM 10500.00	7	1.9%
Educational level	No formal education	0	0.0%
	Primary school education	9	2.4%
	Secondary school education	163	44.1%
	College or University	191	51.6%
	Others	7	1.9%
Planned pregnancy	Yes	163	44.1%
	No	207	55.9%
Patient type	Inpatient	163	44.1%
	Outpatient	207	55.9%
Psychiatry illness	No psychiatry illness	346	93.5%
	Psychotic Disorder/Schizophrenia	2	0.5%
	Bipolar Related Disorders	1	0.3%
	Depressive Disorders	14	3.8%
	Anxiety Disorders	7	1.9%
	Obsessive Compulsive Disorders	0	0.0%
	Trauma-Related Disorders	0	0.0%
	Substance-Related Disorders	0	0.0%
	Neurocognitive Disorders	0	0.0%
	Neurodevelopmental Disorders	0	0.0%
EPDS 12	Yes	31	8.4%
	No	339	91.6%
Intimate partner violence			
Any form of IPV	Yes	237	64.1%
	No	133	35.9%
CB	Yes	202	54.6%
	No	168	45.4%
EV	Yes	111	30.0%
	No	259	70.0%
PV	Yes	9	2.4%
	No	361	97.6%
SV	Yes	13	3.5%
	No	357	96.5%

CB, Controlling behaviour; EV, Emotional violence; PV, Physical violence; SV, Sexual violence.

The prevalence of antenatal depression was 8.4%. IPV was also a significant concern, with 64.1% of the patients reporting some form of IPV. Specifically, 54.6% experienced controlling behaviour, 30.0% experienced emotional violence, 2.4% experienced physical violence, and 3.5% experienced sexual violence.

**Table 2** presents the bivariate analysis of sociodemographic and clinical determinants of antenatal depression among the respondents. A significant difference was found between inpatient and outpatient groups, with depression was more prevalent among inpatients (12.9%) compared to outpatients (4.8%) (χ² = 7.703, p = 0.006). Depression was significantly more common in patients with psychiatric illnesses (25.0%) compared to those without (7.2%) (χ² = 9.237, p = 0.002). Several forms of IPV were significantly associated with depression. Emotional violence was significantly associated with a higher prevalence of depression (17.1% vs. 4.6%, χ² = 15.775, p < 0.001). Physical violence was also significantly associated with depression (55.6% vs. 7.2%, χ² = 26.745, p < 0.001), as was sexual violence (61.5% vs. 6.4%, χ² = 49.601, p < 0.001).

**Table 2:** Bivariate analysis of sociodemographic and clinical determinants of antenatal depression among patients at Hospital Melaka.

Independent Variable	Depression (Yes)		Depression (No)		Total N (%)	Chi-Square	P Value
	N	%	N	%			
	31	8.4%	339	91.6%	370 (100%)		
Age						.431	0.934
18-24	5	10.9%	41	89.1%	46 (12.4%)
25-29	9	7.9%	105	92.1%	114 (30.8%)
30-34	9	8.2%	101	91.8%	110 (29.7%)
35 and above	8	8.0%	92	92.0%	100 (27.0%)
Race						.022	0.882
Malay	27	8.5%	292	91.5%	319 (86.2%)
Non-Malays	4	7.8%	47	92.2%	51 (13.8%)
Marital status						.370	0.543
Married	31	8.5%	335	91.5%	366 (98.9%)
Unmarried	0	0.0%	4	100.0%	4 (1.1%)
Occupation						1.077	0.299
Employed	14	7.0%	186	93.0%	200 (54.1%)
Unemployed	17	10.0%	153	90.0%	170 (45.9%)
Income						.771	0.680
< RM5250.00	22	8.2%	245	91.8%	267 (72.2%)
RM5250.00 – RM11819.00	9	9.4%	87	90.6%	96 (25.9%)
≥ RM 11820.00	0	0.0%	7	100.0%	7 (1.9%)
Educational level						.049	0.825
Secondary Education or Below	15	8.7%	157	91.3%	172 (46.5%)
Post-Secondary Education	16	8.1%	182	91.9%	198 (53.5%)
Planned pregnancy						1.008	0.315
Yes	11	6.7%	152	93.3%	163 (44.1%)
No	20	9.7%	187	90.3%	207 (55.9%)
Patient type						7.703	0.006*
Inpatient	21	12.9%	142	87.1%	163 (44.1%)
Outpatient	10	4.8%	197	95.2%	207 (55.9%)
Psychiatry illness						9.237	0.002*
No Psychiatric Illness	25	7.2%	321	92.8%	346 (93.5%)
Psychiatric Illness	6	25.0%	18	75.0%	24 (6.5%)
Controlling behaviour						3.659	0.056
Yes	22	10.9%	180	89.1%	202 (54.6%)
No	9	5.4%	159	94.6%	168 (45.4%)
Emotional violence						15.775	<0.001*
Yes	19	17.1%	92	82.9%	111 (30.0%)
No	12	4.6%	247	95.4%	259 (70.0%)
Physical violence						26.745	<0.001*
Yes	5	55.6%	4	44.4%	9 (2.4%)
No	26	7.2%	335	92.8	361 (97.6%)
Sexual violence						49.601	<0.001*
Yes	8	61.5%	5	38.5%	13 (3.5%)
No	23	6.4%	334	93.6%	357 (96.5%)

*p < 0.05.

**Table 3** displays the multivariate logistic regression analysis of sociodemographic and clinical determinants of antenatal depression among the respondents. Inpatients had significantly higher odds of depression with an adjusted OR of 0.262 (95% CI: 0.100 to 0.683, p = .006) compared to outpatients. Sexual violence was significantly associated with higher odds of depression, with an adjusted OR of 18.761 (95% CI: 3.603 to 97.684, p <.001) compared to those not experiencing sexual violence.

**Table 3:** Multivariate logistic regression analysis of sociodemographic and clinical determinants of antenatal depression among patients at Hospital Melaka.

Sociodemographic details		EPDS 12		N(%) (370)	Wald χ^2^ statistic		P Value	Adjusted OR (95% CI)
		Yes	No					
Age								
18 - 24		5 (10.9%)	41 (89.1%)	46 (12.4%)	R		
25 - 29		9 (7.9%)	105 (92.1%)	114 (30.8%)	.160		0.690	1.313 (.345 to 4.998)
30 - 34		9 (8.2%)	101 (91.8%)	110 (29.7%)	.077		0.781	1.216 (.307 to 4.815)
35 and above		8 (8.0%)	92 (92.0%)	100 (27.0%)	.170		0.680	1.365 (.311 to 5.989)
Race								
Malay		27 (8.5%)	292 (91.5%)	319 (86.2%)	R		
Non-Malay		4 (7.8%)	47 (92.2%)	51 (13.8%)	1.236		0.266	47 (92.2%)
Occupation								
Employed		14 (7.0%)	186 (93.0%)	200 (54.1%)	R		
Unemployed		17 (10.0%)	153 (90.0%)	170 (45.9%)	.287		0.592	1.312 (.486 to 3.543)
Household income								
< RM 5250.00		22 (8.2%)	245 (91.8%)	267 (72.2%)	R		
RM 5250.00 – RM 11 819.00		9 (9.4%)	87 (90.6%)	96 (25.9%)	.109		0.741	1.228 (.363 to 4.153)
≥ RM 11820.00		0 (0.0%)	7 (100.0%)	7 (1.9%)	.000		0.999	.000 (.000 to -)
Education								
Secondary Education or Below		15 (8.7%)	157 (91.3%)	172 (46.5%)	R		
Post-Secondary Education		16 (8.1%)	182 (91.9%)	198 (53.5%)	.222		0.638	1.252 (.491 to 3.198)
Pregnancy								
Planned pregnancy		11 (6.7%)	152 (93.3%)	163 (44.1%)	R		
Unplanned pregnancy		20 (9.7%)	187 (90.3%)	207 (55.9%)	1.835		0.176	1.883 (.754 to 4.703)
Patient type								
Inpatient		21 (12.9%)	142 (87.1%)	163 (44.1%)	R		
Outpatient		10 (4.8%)	197 (95.2%)	207 (55.9%)	7.496		0.006*	.262 (0.100 to 0.683)
Psychiatry illness								
No psychiatric illness		25 (7.2%)	321 (92.8%)	346 (93.5%)	R		
Psychiatric Illness Present		6 (25.0%)	18 (75.0%)	24 (6.5%)	2.017		0.156	2.481 (.708 to 8.693)
Intimate partner violence								
Controlling Behaviour	Yes	22 (10.9%)	180 (89.1%)	202 (54.6%)	.002		0.960	1.024 (.399 to 2.625)
	No	9 (5.4%)	159 (94.6%)	168 (45.4%)	R		
Emotional Violence	Yes	19 (17.1%)	92 (82.9%)	111 (30.0%)	2.356		0.125	2.075 (.817 to 5.269)
	No	12 (4.6%)	247 (95.4%)	259 (70.0%)	R		
Physical Violence	Yes	5 (55.6%)	4 (44.4%)	9 (2.4%)	.220		0.639	1.563 (.242 to 10.110)
	No	26 (7.2%)	335 (92.8%)	361 (97.6%)	R		
Sexual Violence	Yes	8 (61.5%)	5 (38.5%)	13 (3.5%)	12.128		<0.001*	18.761 (3.603 to 97.684)
	No	23 (6.4%)	334 (93.6%)	357 (96.5%)	R		

*p <0.05.

## 4 Discussion

This study aimed to investigate the relationship between IPV and antenatal depression among pregnant women at Hospital Melaka. We hypothesized that women experiencing IPV would be more likely to develop antenatal depression. Our findings support this hypothesis, demonstrating significant associations between various forms of IPV—specifically sexual and physical violence—and the prevalence of antenatal depression. In addition to IPV, we explored other sociodemographic factors and pregnancy-related complications that may contribute to antenatal depression. The discussion below addresses these findings holistically, considering the overall prevalence of antenatal depression and IPV, the associations observed, and how cultural factors may have influenced our results.

To our knowledge, this is the first study to investigate the relationship between antenatal depression and IPV in a population from Melaka. The prevalence of antenatal depression in our study (8.4%) was similar to the prevalence reported in a previous cross-sectional study conducted in a hospital in Ipoh, Perak (8.6%). However, our finding was lower than those reported in studies from the east coast of Peninsular Malaysia (12.2%) and Penang (20%). The slightly lower prevalence maybe attributed to cultural factors such as the fear of stigmatization and reluctance to disclose depressive symptoms, despite efforts to ensure privacy during interviews (19). This highlights the importance of considering both social and cultural dynamics when studying antenatal depression. While our findings align with previous research, they also emphasize the potential for underreporting due to societal attitudes towards mental healthOur study found a high prevalence of IPV among pregnant women, with 64.1% of the participants reporting experiencing some form of IPVThis figure is significantly higher than the IPV prevalence reported in previous studies conducted in Malaysia, where prevalence rates ranged from 4.94% to 35.9%. This difference may be explained by the broader scope of IPV assessed in our study, which included controlling behaviour as part of the IPV assessment. In contrast, other studies in the systematic review primarily focused on emotional, physical, and sexual violence without addressing controlling behaviour (20, 21). The rest also only concentrated on physical and sexual violence (22, 23).

The most commonly reported forms of IPV in our study were controlling behavior (54.6%) and emotional violence (30%). This aligns with the existing literature, which identifies psychological abuse as the most prevalent form of IPV during pregnancy (21). The high prevalence of controlling behavior and emotional violence in our study underscores the pervasive nature of psychological abuse during pregnancy, often overlooked in discussions of IPV but has significant mental health consequences.

The elevated prevalence of IPV observed in this study underscores the urgent need for healthcare providers to routinely screen for IPV, particularly in antenatal settings, where women may feel more comfortable seeking help. Numerous studies have found a strong association between IPV and antenatal depression (24), and our findings are consistent with this body of research. Our study identified significant associations between emotional, physical, and sexual violence and antenatal depression, although the strength of these associations varied.

Notably, sexual violence had the strongest correlation with antenatal depression, with women who experienced sexual IPV being 18 times more likely to develop depression. This finding aligns with research that underscores the profound psychological trauma linked to sexual violence, especially during pregnancy, a particularly vulnerable period for women (25, 26). Sexual violence, as one of the most severe forms of IPV, may be more readily reported due to its clear violation of personal boundaries and its legal status as a crime (27).

Physical violence, although less commonly reported, also showed a strong association with antenatal depression, supporting previous research that indicates physical harm significantly impacts mental health during pregnancy (4). Women subjected to physical violence may experience increased fear, anxiety, and feelings of helplessness, all of which contribute to depressive symptoms.

However, emotional violence exhibited a weaker association with antenatal depression in our study. One explanation for this could be the cultural normalization of emotional abuse in Malaysian society. In some Asian contexts, behaviors considered emotionally abusive may be overlooked or not recognized as forms of abuse (28). This normalization can lead women to underreport emotional violence or fail to attribute their emotional distress to their partner’s behavior. Additionally, strong family support systems, which are common in Malaysia, may buffer the effects of emotional violence, reducing its impact on mental health (4, 29).

Cultural factors also likely influenced the reporting of antenatal depression in our study. The stigma surrounding mental health in Malaysia, particularly in Melaka, may have led to underreporting of depressive symptoms due to societal expectations of resilience during pregnancy (19). Women may have minimized or failed to recognize their symptoms, resulting in an underestimation of the true prevalence of antenatal depression. Despite the use of culturally validated tools and efforts to ensure confidentiality, these factors may have limited the accurate reporting of both IPV and depression. Future research should employ mixed-method approaches to better understand how cultural norms influence mental health reporting among pregnant women in Malaysia.

In addition to IPV, we identified other factors associated with antenatal depression, particularly among women who were admitted to the inpatient wards. These women may have experienced more severe pregnancy complications, such as preterm labor, gestational diabetes, or preeclampsia, which can increase stress and anxiety, contributing to depressive symptoms. The emotional toll of extended hospital stays, coupled with concerns about the health of both the mother and fetus, may lead to feelings of helplessness, exacerbating depressive symptoms.

Our findings suggest that the inpatient setting can create an environment where women feel more secure in discussing sensitive issues, such as depression or IPV. The privacy provided by hospital wards may reduce the fear of immediate repercussions from family members or partners, allowing women to disclose symptoms more freely (21). This may explain why women in our study who were inpatients more likely to report depressive symptoms compared to outpatient.

Interestingly, while these pregnancy complications were linked to higher rates of depression, no other sociodemographic factors in our study showed a significant association with antenatal depression. This may reflect broader social changes, such as the increasing empowerment of women in Malaysia and greater awareness of mental health issues. As more women enter the workforce and gain access to education and healthcare services, they may be better equipped to manage the psychological stressors of pregnancy, reducing the risk of antenatal depression (30).

Our results suggest that while IPV remains a significant predictor of antenatal depression, pregnancy-related complications such as inpatient admissions may also contribute to a woman’s mental health status. Understanding these additional risk factors is important for developing holistic interventions that address not only IPV but also the broader psychosocial challenges faced by pregnant women.

One of the key strengths of this study is the high response rate of 96.4%, enhancing the generalizability of the findings to pregnant women in Melaka. The study is among the first to explore the relationship between IPV and antenatal depression in this population, offering valuable insights into local antenatal mental health. The use of validated tools, such as the EPDS and WHO Women’s Health and Life Experiences Questionnaire, strengthens the reliability of the findings. Furthermore, the inclusion of both inpatient and outpatient participants provides a more comprehensive view of the factors influencing antenatal depression, allowing for a better understanding of pregnancy-related complications in this context.

However, several limitations should be considered when interpreting these results. First, the cross-sectional design of the study does not allow for the establishment of causality between IPV and antenatal depression. Since data on IPV and antenatal depression were collected simultaneously, it remains unclear whether IPV leads to depression or if women experiencing depression are more vulnerable to IPV. To address this limitation, we employed multivariate logistic regression to adjust for potential confounders. However, we recognize that residual confounding may still exist, and these results should be interpreted as associations rather than definitive cause-and-effect relationships.

Another limitation is the use of convenience sampling, which may introduce selection bias. Women who attended the hospital for antenatal care may not represent the broader population of pregnant women, particularly those who do not seek regular medical care. This could have influenced the prevalence rates of both IPV and antenatal depression in our sample.

Cultural factors may have also played a role in underreporting, particularly in relation to emotional violence and depressive symptoms. Despite our efforts to ensure privacy and confidentiality during data collection, some participants may have been hesitant to disclose their experiences due to the stigma surrounding both IPV and mental health in Malaysia. This could have led to an underestimation of the true prevalence of antenatal depression in our study.

In summary, while this study provides important insights into the relationship between IPV and antenatal depression, it also highlights the need for further research to explore these issues in greater depth. Future studies should consider using longitudinal designs and mixed-method approaches to better understand the cultural and temporal dynamics of IPV and antenatal mental health outcomes.

## 5 Conclusion

The prevalence of antenatal depression using EPDS was 8.4% in Hospital Melaka. Experiencing sexual violence increased the odds of antenatal depression by 18 times. Being an inpatient also increased the odds of antenatal depression. Healthcare workers should be trained to recognise possible risk factors for antenatal depression, especially sensitive issues like IPV.

## Data Availability

The original contributions presented in the study are included in the article/supplementary material. Further inquiries can be directed to the corresponding author.
